# Protecting patients and staff in residential treatment centers during exposure to COVID-19: commentary

**DOI:** 10.1186/s13722-021-00258-2

**Published:** 2021-07-30

**Authors:** Kimberly A. Johnson, Carolyn Keough, Holly Hills, Wouter Vermeer, Rebecca Lengnick-Hall, Moira McNulty, Mark McGovern, Hendricks Brown

**Affiliations:** 1 College of Behavioral and Community Science, Department of Mental Health Law and Policy, University of South Florida, Tampa, FL USA; 2 Operation PAR Inc, Clearwater, FL USA; 3 Department of Psychiatry and Behavioral Sciences, Feinberg School of Medicine, Northwestern University, Chicago, IL USA; 4 The Brown School, Washington University in St. Louis, St. Louis, MO USA; 5 Department of Medicine, Section of Infectious Diseases, University of Chicago, Chicago, IL USA; 6 Center for Behavioral Health Services and Implementation Research, Department of Psychiatry and Behavioral Sciences, Stanford University School of Medicine, Palo Alto, CA USA; 7 Division of Primary Care and Population Health, Department of Medicine, Stanford University School of Medicine, Palo Alto, CA USA

## Abstract

**Background:**

The COVID-19 pandemic has created a crisis in access to addiction treatment. Programs with residential components have been particularly impacted as they try to keep infection from spreading in facilities and contributing to further community spread of the virus. This crisis highlights the ongoing daily trade-offs that organizations must weigh as they balance the risks and benefits of individual patients with those of the group of patients, staff and the community they serve.

**Main body:**

The COVID-19 pandemic has forced provider organizations to make individual facility level decisions about how to manage patients who are COVID-19 positive while protecting other patients, staff and the community. While guidance documents from federal, state, and trade groups aimed to support such decision making, they often lagged pandemic dynamics, and provided too little detail to translate into front line decision making. In the context of incomplete knowledge to make informed decisions, we present a way to integrate guidelines and local data into the decision process and discuss the ethical dilemmas faced by provider organizations in preventing infections and responding to COVID positive patients or staff.

**Conclusion and commentary:**

Provider organizations need decision support on managing the risk of COVID-19 positive patients in their milieu. While useful, guidance documents may not be capable of providing support with the nuance that local data and simulation modeling may be able to provide.

## Background

The COVID-19 pandemic created a crisis in access to addiction treatment as evidenced by the increase in overdose deaths, news stories about treatment program closures, and reduced capacity in those that remain open [1]. Many addiction treatment agencies established protocols to reduce the risk of exposing patients and staff. Most commonly instituted were symptom and temperature checks at the door, requirements of masks and physical distancing, and limited or no visitor policies [2]. These changes required significant alterations to standard treatment protocols, such as group therapy, that require person to person interaction. With subsiding levels of community spread, providers are now confronted with questions regarding which protocols they can reasonably maintain. While trade associations, the CDC, and states have recommended that treatment organizations get staff vaccinated [3] and the recent district court ruling allows providers to require staff to be vaccinated [4], workers’ hesitancy and labor shortages may prevent treatment programs from enforcing vaccine mandates. Surveys of people with substance use disorders indicate their vaccine hesitancy mirrors the general public with about half expressing hesitancy [5]. In trying to manage these complex issues, provider organizations face an ethical dilemma that pits the rights of an individual staff member or patient against the needs of the rest of the patients, the organization and the community as a whole.

## Organization-level decision process

To illustrate this issue, we present an example of the experience of a detoxification program (ASAM level 3.7), as it highlights the multiple layers of an organization’s decision process and the tradeoffs made to balance the needs of an infected patient, the other patients, staff, the community, and the organization. In the example, the patient was referred to the detoxification program on an involuntary commitment for withdrawal from alcohol. The patient presented as asymptomatic for COVID-19 (negative response to screening questions and no fever) and was awaiting test results. The patient was admitted using standard admission protocols including the COVID-19 era protocol of being placed in a room alone. Several hours after admission, despite a lack of symptoms, the test came back positive for COVID-19.

The agency had to make a decision about what to do with the patient that involved multilevel ethical, legal and financial aspects that all had to be weighed. What was in the best interest of the patient who was in active withdrawal from alcohol? What was in the best interest of the other patients and the staff in the facility? What were the financial ramifications of these decisions on an organization already strapped for cash due to reduced capacity? What were the legal liabilities of any decision made? Beyond the immediate crisis, what would be the impact of the decisions to the organization and the community within which it operates?

Some organizations have made decisions based purely on a financial basis and closed programs that cannot make money at reduced capacity. This creates upstream problems for systems managers and communities that need to find alternatives. Other organizations have tried to find a balance based on the pillars of medical ethics [6]. The concept of beneficence (the duty to do good) and non-maleficence (the duty to do no harm) as well as respect for autonomy and the duty of ensuring justice (treating all people equitably) are generally thought of as concepts that are to be addressed on an individual basis. During acute situations, such as a pandemic, we need to expand these concepts from the individual patient into the community of people within which s/he resides.

Medical ethics has not done well addressing the conflict of rights between a group or community and the individual [7]. This type of moral conflict commonly occurs in addiction treatment programs and the challenges posed have been further exacerbated in the context of the pandemic. Under such complex scenarios, making the best decision quickly is not easy, and most decision makers will weigh one aspect or another more heavily. Guidance from government or professional associations can be helpful, but may not be timely or adequately nuanced to address specific local scenarios.

In our example, the agency did consult with the local Department of Health, and determined that the best place for the patient was at the detoxification center. The patient did not require hospitalization and no other location could manage the patient’s withdrawal. The decision to do what was best for the individual patient created a cascade of additional decisions that the agency had to make concerning this patient, staff, other patients, and the community.

Specifically, the patient was retained for the period of detoxification, five days, with treatment as normalized as possible with minimal contact with staff and no contact with other patients. After five days the patient was released to a quarantine and isolation facility designed to house COVID-19 positive patients who are homeless and not sick enough to be hospitalized, thereby protecting the public. To decrease transmission risk to other patients, a thorough deep cleaning was conducted in all public areas, the COVID-19 positive patient was isolated on a separate wing, and patients were informed that there was a patient who had tested positive in the facility. To protect staff in situations where close contact was required (bathroom trips, medical assessment), staff wore full personal protective equipment (PPE) including N95 masks and face shields. However, where direct contact could be avoided, such as case management and counseling, work was conducted by phone. These choices were made during a time where N95 masks were scarce and knowledge about how the virus was transmitted was incomplete.

Our example highlights just one way in which even following preventative guidelines can provide less than complete protection, and many other risks can occur. In this era of incomplete vaccination and removal of mask restrictions, staff, rather than the patients, could be a source of spread of infection. This has been particularly evident in nursing home settings [8] and has occurred in residential treatment programs as well [9], especially when there no protocol for testing staff on a routine basis. Like patients, infected staff can often present without symptoms. The risk of spread to other staff and patients is high under either the staff or patient transmission scenario. Vaccination hesitancy of both staff and patients means that there remains an ongoing risk, even late in the pandemic.

Given that screening is imperfect, vaccination rates are lower than anticipated, and new variants open the door to continued infections, agencies need to plan on how to manage patients and staff who are positive. How they make that plan will depend on the state and local legal and funding environment, patient treatment needs, facility structure, layout and staffing, and organization mission and values. Typically, treatment agencies must assign weights informally to the various elements and stakeholders needs without the ability to accurately predict the outcomes of alternative actions. Clear guidance that is based on scientific principles is needed to ensure treatment access and continuity. Because standard research methods like randomized trials are too protracted and expensive to provide all the information that organizations need moment by moment in a crisis, and guidance documents are often unable to anticipate all the variations of local scenarios, tools that use local data to conduct scenario planning might improve the ability to compare the likely outcomes of alternative strategies. Simulation modeling tools that can predict likely outcomes are used widely in other industries [10, 11].

## Conclusion and commentary

In our example scenario, the agency responded quickly and further spread was prevented. The crisis allowed the organization to better prepare for future situations that were likely to arise. Indeed, a few months later a staff person exposed the whole facility which then by default became a COVID-19 quarantine ward for two weeks with no new admissions. Both similar, and more dire scenarios where entire patient populations have become infected and ill, have happened across the country. Despite measures to enhance access to telehealth and loosened regulation of medication management, preliminary estimates indicate that treatment utilization decreased in 2020 [12] and that those accessing care were a less severe, more well connected group [13]. What is more, overdose deaths increased dramatically [14], suggesting that decision making under these complex circumstance is both hard and sub-optimal.

We note that these ethical challenges are universal and will continue to reappear throughout every stage of this pandemic. There is a need to use empirical data to advance general principles.

Quality improvement monitoring, timely reporting, and analysis of data on patients (e.g., transmission) in different contexts (e.g. level of isolation) are necessary to ascertain which accommodations affect outcomes, and consequently inform future treatment protocols. In addition, local decision making needs to take into account local conditions. Just as tools have been developed to help clinicians make better decisions for single complex patient presentations [15], the necessity of tools to support complex management and community health decisions is arising. Decision modeling tools, when informed by timely local data (e.g., community prevalence, vaccination rates), may help systems of care as well as individual agencies to weigh predicted effects on individual patients, residents and staff, and the organization and community. Comparing their respective risks and benefits, as well as individual rights, freedoms, and sacrifices to the common good would make clear the trade-offs of the available mitigation protocols on different stakeholders within a medical ethics framework, and remove from consideration those that provide unacceptable risk for individuals, the group, or the community. Complex, multilevel, life and death decisions should be informed by such examinations that allow stakeholders to make their ethical choices explicit. It is time we adapt the tools we have used to support scenario planning for other situations to this critical health issue.

## Data Availability

No data or materials are included with this paper.
